# Some Biochemical and Hematological Parameters among Petrol Station Attendants: A Comparative Study

**DOI:** 10.1155/2015/418724

**Published:** 2015-11-08

**Authors:** Hala Samir Abou-ElWafa, Ahmed A. Albadry, Abdel-Hady El-Gilany, Fagr B. Bazeed

**Affiliations:** ^1^Public Health & Community Medicine Department, Faculty of Medicine, Mansoura University, Mansoura 35516, Egypt; ^2^Department of Medical Biochemistry, Faculty of Medicine, Mansoura University, Mansoura 35516, Egypt

## Abstract

*Objective*. To describe selected biochemical and hematological parameters (blood picture, liver enzymes, and kidney functions) in petrol station attendants in Mansoura city. *Methods*. This is a comparative cross-sectional study. The exposed group included 102 petrol station attendants. They were compared to a matched group of healthy 102 male service and office workers at the Faculty of Medicine, Mansoura University. The results of blood picture, liver enzymes, and kidney functions were compared between both groups. *Results*. Mean Red Blood Cells (RBCs) count, hemoglobin level, and Hematocrit (HCT) level were significantly lower in petrol station attendants than the comparison group. All other blood picture parameters showed nonsignificant difference between both groups. Liver enzymes, renal functions, serum albumin, and total protein showed statistically nonsignificant difference between both groups except for alanine aminotransferase (ALT) which was significantly higher in petrol station attendants. *Conclusions*. Some laboratory parameters among petrol station attendants showed changes that could be attributed to workplace exposure and should be given attention at preemployment and periodic medical examination.

## 1. Introduction

Petrol (or gasoline) is a mixture of volatile hydrocarbons, while diesel fuel is a distillate of petroleum which contains paraffins, alkenes, and aromatics [1].

Fuel (petrol and diesel) filling station attendants (FFSAs) are exposed to a mixture of hydrocarbons in fuel vapours during dispensing fuel and to the gases from vehicular exhaust [2]. In filling stations, the volume of fuel dispensed as well as the ambient temperature contributes significantly to the increased emission of volatile hydrocarbons. Benzene (BZ) could be considered to be the most hazardous; xylene and toluene have toxicities in line with other aromatics of lower/different concern compared to BZ [3].

Certain people have a greater risk of exposure to gasoline vapors; these include filling station workers, service station attendants, drivers of gasoline trucks, and refinery workers. The volatile nature of petrol products makes them readily available in the atmosphere any time it is dispensed, especially at petrol filling stations and depots [4].

Many of the harmful effects seen after exposure to gasoline are due to the individual chemicals in the gasoline mixture, such as benzene, lead, and oxygenates. Breathing small amounts of gasoline vapors can lead to nose and throat irritation, headaches, dizziness, nausea, vomiting, confusion, and breathing difficulties. Some effects of skin contact with gasoline include rash, redness, and swelling. Allergic reactions (hypersensitivity) have been reported but these are rare occurrence [5, 6]. The adverse health effects of gasoline exposure may be primarily related to impairment of the haemopoietic system with bone marrow depression [7].

Occupational diseases in gasoline filling workers have been recognized for many years and affect workers in different ways; such diseases are still problems in all parts of the world. The numbers of such work-related diseases in developing countries are much higher in reality than the numbers that are reported. The numbers of cases and types of occupational diseases are increasing in both developing and industrialized countries [8].

To the best of the authors' knowledge no past studies have investigated the laboratory profile of petrol station attendants as a result of their workplace occupational exposure in Egypt.

The objective of this study was to describe selected biochemical and hematological parameters (blood picture, liver enzymes, and kidney functions) in petrol station attendants in Mansoura city.

## 2. Methods

A comparative cross-sectional study was conducted upon petrol station attendants at Mansoura city during the period from January 1, 2015, to March 31, 2015.

### 2.1. Subjects

#### 2.1.1. The Study Group

There are 14 petrol stations in Mansoura city and neighborhood as detected by “*Google* Earth.” The study included 102 out of 112 petrol station attendants with a response rate of 91%, recruited from 6 stations with the largest workforce (more than 5 workers) and agreed to participate in the study. All attendants are males working either in fuelling service for 24 hours/day for three days in one week and for four days in the next week in an alternating pattern or in car maintenance and wash and oiling services for 8 hours/day from 9 a.m. to 5 p.m.

The petrol station attendants were interviewed and a blood sample was taken at the administrative office at each station at 10 a.m. where their workload at this time is relatively light.

#### 2.1.2. The Comparison Group

The group comprised 102 male service and office workers at the Faculty of Medicine, Mansoura University, comparable to petrol station attendants in most of the variables except for the risk of exposure to petrol. They represent 89.5% of all male service and office workers. They were interviewed and examined at the department of public health and community medicine during the work day.

*Ethical Consideration*. Approvals of the studied petrol stations, Faculty of Medicine authorities, and the research ethics committee were obtained. An informed verbal consent of study subjects to participate voluntarily in the study with a full right to withdraw, as they were willing to make a health check for free, was obtained with assurance of confidentiality and anonymity of the data.

Each participant was subjected to the following:

1. Interview: a questionnaire was used to collect the following information: sociodemographic and occupational profile of workers; usage of personal protective equipment and reasons for nonusage; general health status; and respiratory complaints.
2. Laboratory investigation: a 5 mL blood sample was taken from each participant through venipuncture which was then divided into the following:
  - There is two mL blood collected in a plastic tube containing ethylenediaminetetraacetic acid (EDTA) for complete blood picture.
  - There is three mL blood collected in a dry plastic tube for kidney function tests (including urea, uric acid, and creatinine) and liver function tests (including ALT, aspartate aminotransferase (AST), serum albumin, and total protein). The sample was allowed to clot naturally to separate the serum for analysis and was stored upright at room temperature until it was transported to the laboratory for analysis. In the laboratory, each sample was centrifuged and stored in the freezer at −70°C until being processed.

*Data Analysis*. Data were entered and statistically analyzed using the Statistical Package for Social Sciences (SPSS) version 17. The cut-off points for different parameters were defined according to the laboratory standard defined by the manufacturer of the kits. Qualitative data were described as numbers and percentages. Chi-square (*χ* ^2^) test was used for comparison between groups. Quantitative data were described as means (standard deviation (SD)) or medians, as appropriate. They were tested for normality by Kolmogorov-Smirnov test. In the normally distributed variables, independent sample *t*-test was used, while in nonnormally distributed variables, Mann Whitney test was used for comparison between groups. “*p* value ≤ 0.05” was considered to be statistically significant.

## 3. Results

Petrol station attendants matched the comparison group in all sociodemographic and occupational profile items except for the median duration of smoking which was longer in petrol station attendants (Table 1).

Mean RBCs count, hemoglobin level and the percent anemic, and HCT level were significantly lower in petrol station attendants than the comparison group. All other blood picture parameters showed nonsignificant difference between both groups (Table 2).

Liver enzymes, renal functions, serum albumin, and total protein (both the mean level and the percentage of abnormal levels) showed statistically nonsignificant difference between both groups except for ALT which was significantly higher in petrol station attendants (Table 3).

Both groups neither had health insurance, worn personal protective equipment (PPE) due to their unavailability nor had preemployment or periodic examination. Among petrol station attendants, one (1%) complained of headache and vertigo, one (1%) complained of morning cough and shortness of breath, and two (2%) complained of skin itch, redness, and rash (data are not shown in tables).

## 4. Discussion

The association between exposure to benzene or benzene-containing mixtures and certain types of blood disorders has been shown in epidemiological studies in different countries [9, 10]. Benzene, an important component of petrol, is a widely distributed environmental contaminant [11] and has mainly been associated with increased incidence of blood disorders [12].

The results of the study showed that the mean hemoglobin level and RBCs count of petrol station attendants were significantly lower than those of the comparison group while mean white blood cells (WBCs) and platelets counts were higher among petrol station attendants with nonsignificant difference between both groups. Similarly, in hematological assessment of gasoline exposure among petrol filling workers in Baghdad, their mean hemoglobin level, WBCs, and RBCs counts were significantly lower than those of comparison group [7].

In this study, mean HCT value was significantly lower in petrol station attendants than comparison group while mean corpuscular volume (MCV), mean corpuscular hemoglobin (MCH), and mean corpuscular hemoglobin concentration (MCHC) were similar in both groups with statistically nonsignificant difference between them. Previous studies did not detect decreased blood cell counts on routine monitoring of workers exposed to low level of benzene [13, 14].

Liquefied petroleum gas- (LPG-) exposed workers of Gaza governorates were found to have significantly higher values of RBC, hemoglobin, HCT, MCH, MCHC, and platelets than comparison group while the mean WBCs count was significantly lower. This study showed a significant effect of LPG exposure on the haematological parameters of LPG workers compared to comparison group [15]. Their results agree with the results of two studies of subjects exposed to natural gas [16, 17].

In the study, the median values of ALT and AST were higher among petrol station attendants than the comparison group where ALT showed significant difference between them while AST showed nonsignificant difference. Similarly, the mean values of serum AST and ALT were significantly higher among LPG workers of Gaza governorates (34.7 ± 5.39 U/L and 32.7 ± 4.30 U/L, resp.) compared with controls (16.6 ± 4.48 U/L and 14.6 ± 4.15 U/L, resp.) [15]. These results also agree with the results obtained from other settings for LPG or natural gas exposures. Hu et al. [18] showed that long term exposure to coke oven emissions increased the risk of liver dysfunction, while Chen et al. [19] and Wu et al. [20] explored the dose-response relationship between exposure to natural gas emissions in coke oven workers and the elevation of some serum liver enzymes and reported a significant elevation of some liver enzymes in these workers that may have been related to their exposure to natural gas. Abnormal liver functions alongside neurological symptoms were reported in a case report of a male, following accidental inhalation of natural gas containing propane and butane [21].

This study showed that serum uric acid, urea, and creatinine levels were higher among petrol station attendants than comparison group however statistically nonsignificant. Similarly, serum levels of urea and creatinine were shown to be significantly elevated in gasoline filling workers in Sulaimani City, Kurdistan, more than comparison group, though these values are still within the highest accepted normal ranges [22]. Also, the study of LPG workers of Gaza governorates showed that urea, creatinine, and uric acid levels were significantly higher in workers compared with controls [15]. However, Viau et al. [23] did not find significant effects on kidney function markers of refinery workers who were occupationally exposed to hydrocarbons, the major component of natural or LPG gas.

This study showed lower mean values of serum albumin and total protein among petrol station attendants than comparison group with statistically nonsignificant difference between them. In Sulaimani City, Kurdistan, total plasma protein levels were not significantly changed in gasoline filling station workers compared to nonexposed subjects, while plasma albumin levels were significantly elevated in workers more than comparison group [22].

Fuel products are mixtures of aliphatic and aromatic hydrocarbons mostly related to gasoline; most of them are toxic to many organ systems including the kidney [24, 25], which may be attributed to an increase in liberating toxic metabolites including reactive oxygen species. While experiments with rats indicate that exposure by inhalation to the aromatic hydrocarbons toluene, styrene, and xylene was nephrotoxic [26], this effect has not been confirmed in man [27]. Both human and experimental studies suggest that many chemicals can affect the kidney [28]. Gasoline includes many chemicals and additives where anyone could be the cause for such deterioration in renal functions. Accordingly, the identification of specific solvents and exposed job categories at risk would improve intervention to prevent or delay mild renal impairment progression to end-stage renal disease in the occupational setting [22].

In this study, 1% of petrol station attendants complained of headache and vertigo, morning cough, and shortness of breath, and 2% complained of skin itch, redness, and rash. The results of the study of workers at filling and distribution stations of LPG of Gaza governorates showed significantly higher rates of nearly all health status items in LPG workers than comparison group, especially experiencing headache or fatigue in work, eye itches, redness, pain, skin itches, redness and rash, and respiratory complaints, such as fatigue during climbing stairs, repeated sneezing during working hours, shortness of breath in poorly ventilated room, and shortness of breath in the workplace [15].

Studies in different settings showed similar results to that of Gaza Strip, especially concerning health complaints related to respiratory system [29, 30]. These health-related complaints of LPG workers are likely to be due to the pharmacological effect of LPG. Inhalation of gaseous propane, which is the major component of LPG, is known to cause dizziness, nausea, vomiting, confusion, hallucinations, and a feeling of euphoria [31] and to suppress central nervous system (CNS) function [32].

The frequency of general health and respiratory complaints among petrol station attendants in our study is low despite the nonuse of PPE at work and this could be attributed to the outdoor location of petrol stations together with the enclosed system for fuelling vehicles that could minimize exposure to benzene.

## Figures and Tables

**Table 1:** Sociodemographic and occupational profiles of study groups.

Characteristic	Petrol station attendants (102)		Comparison group (102)		Test of significance
Age (years) Mean ± SD	37.6 ± 12.1		37.4 ± 10.1		*t* = 0.12, *p* = 0.9

	Number	%	Number	%	

Education					
Illiterate/read and write	17	16.7	10	9.8	*χ* ^2^ = 2.1, *p* = 0.15
Primary and above	85	83.3	92	90.2	

Marital status					
Unmarried	21	20.6	16	15.7	*χ* ^2^ = 0.8, *p* = 0.4
Married	81	79.4	86	84.3	

Residence					
Rural	76	74.5	80	78.4	*χ* ^2^ = 0.4, *p* = 0.5
Urban	26	25.5	22	21.6	

Type of contract					
Temporary	85	83.3	81	79.4	*χ* ^2^ = 0.5, *p* = 0.5
Permanent	17	16.7	21	20.6	

Duration of employment (ys) Median (min–max)	10 (0.1–40)		6.5 (0.4–33)		*Z* ^*∗*^ = 1.8, *p* = 0.08

Current smoking	46	45.1	33	32.4	*χ* ^2^ = 3.5, *p* = 0.06

Duration of smoking (ys)^#^ Median (min–max)	10 (1.5–40)		6 (2–21)		*Z* ^*∗*^ = 2.4, *p* = 0.02

SD = standard deviation.

^*∗*^ *Z* of Mann Whitney test.

^#^Among smokers.

**Table 2:** Complete blood picture of the study groups.

Parameter	Petrol station attendants (102)	Comparison group (102)	Test of significance
Platelets (×10^9^/L)	252.3 ± 58.4	243.5 ± 46.9	*t* = 1.2, *p* = 0.2
WBCs (×10^9^/L)	7.2 ± 2.2	6.9 ± 1.7	*t* = 1.1, *p* = 0.3
RBCs (million cells/mcL)	4.8 ± 0.4	5.3 ± 0.5	*t* = 7.9, *p* ≤ 0.001
Hemoglobin (gm/dL)	13.9 ± 1.3	15.2 ± 1.2	*t* = 7.4, *p* ≤ 0.001
<12 gm/dL	7 (6.9)	1 (0.98)	*p* = 0.06
≥12 gm/dL	95 (93.1)	101 (99.02)	
HCT (%)	39.7 ± 3.4	43.7 ± 3.6	*t* = 8.2, *p* ≤ 0.001
MCV (femtoliter)	82.7 ± 6.4	82.4 ± 4.3	*t* = 0.4, *p* = 0.7
MCH (pg/cell)	28.6 ± 2.6	28.7 ± 1.9	*t* = 0.3, *p* = 0.8
MCHC (gm/dL)	34.7 ± 1.3	34.9 ± 0.9	*t* = 1.3, *p* = 0.2

Cells/mcL = cells per microliter, gm/dL = grams per deciliter, and pg/cell = picograms per cell.

**Table 3:** Liver enzymes, renal functions, serum albumin, and total protein of the study groups.

Laboratory parameter	Petrol station attendants (102)	Comparison group (102)	Test of significance
ALT (U/L)	30 (12–121)	26 (11–125)	*Z* = 2.8, *p* = 0.004
Median (min–max)			
>45 U/L	12 (11.8)	12 (11.8)	—
≤45 U/L	90 (88.2)	90 (88.2)	

AST (U/L)	31 (14–154)	30.5 (19–115)	*Z* = 0.3, *p* = 0.8
Median (min–max)			
>40 U/L	18 (17.6)	17 (16.7)	*p* = 0.9
≤40 U/L	84 (82.4)	85 (83.3)	

Uric acid (mg/dL)	4.5 (0.7–7)	4.2 (2.2–34)	*Z* = 1.9, *p* = 0.06
Median (min–max)			
>6.0 mg/dL	8 (7.8)	8 (7.8)	—
≤6.0 mg/dL	94 (92.2)	94 (92.2)	

Creatinine (mg/dL)	0.9 ± 0.4	0.8 ± 0.4	*t* = 1.8, *p* = 0.08
Mean ± SD			
>1.3 mg/dL	3 (2.9)	1 (0.98)	*p* = 0.6
≤1.3 mg/dL	99 (97.1)	101 (99.02)	

Urea (mg/dL)	28.4 ± 8.1	26.6 ± 5.6	*t* = 1.8, *p* = 0.07
Mean ± SD			
>50 mg/dL	1 (0.98)	0 (0.0)	*p* = 1
≤50 mg/dL	101 (99.02)	102 (100.0)	

Serum albumin (gm/dL)	4.2 ± 0.4	4.3 ± 0.4	*t* = 1.8, *p* = 0.08
Mean ± SD			
<3.5 gm/dL	1 (0.98)	1 (0.98)	—
≥3.5 gm/dL	101 (99.02)	101 (99.02)	

Total protein (gm/dL)	7.1 ± 0.9	7.3 ± 0.6	*t* = 1.9, *p* = 0.06
Mean ± SD			
<6.0 gm/dL	3 (2.9)	0 (0.0)	*p* = 0.2
≥6.0 gm/dL	99 (97.1)	102 (100.0)	

U/L = units per liter.

## Study Limitations

This study suffers some limitations. There were no records for baseline and periodic medical examination of workers to identify changes that could be attributed to petrol exposure. Being a small-scale study limits the generalization of results to the total population of petrol station attendants. Also there was no environmental and personal monitoring for petrol exposure to be related to the laboratory changes. The health hazards could be underestimated as petrol attendants who experienced serious hazards may had been exempted from work or changed it.

## Recommendations

There should be the introduction of initial medical or laboratory testing for petrol station attendants before being hired to their jobs to check their fitness and suitability for duties at petrol stations. A medical observation including preemployment and periodic medical checkup for early recognition and removal of sensitive workers from their working places before development of chronic impairment should be done. The availability and use of PPE at work to minimize workplace petrol exposure should be encouraged. Further longer term perspective studies of petrol workers help to get a more comprehensive picture of long term effects of petrol exposure.
